# Role of endothelin receptor antagonist; bosentan in cisplatin-induced nephrotoxicity in ovariectomized estradiol treated rats

**DOI:** 10.12860/jnp.2015.25

**Published:** 2015-10-01

**Authors:** Alieh Zahedi, Mehdi Nematbakhsh, Maryam Moeini, Ardeshir Talebi

**Affiliations:** ^1^Water & Electrolytes Research Center, Isfahan University of Medical Sciences, Isfahan, Iran; ^2^Department of Physiology, School of Medicine, Isfahan University of Medical Sciences, Isfahan, Iran; ^3^Isfahan MN Institute of Basic & Applied Sciences Research, Isfahan, Iran; ^4^Department of Clinical Pathology, School of Medicine, Isfahan University of Medical Sciences, Isfahan, Iran

**Keywords:** Cisplatin, Estradiol, Nephrotoxicity, Bosentan, Rat

## Abstract

*Background: * Endothelin-1 (ET-1) is a vasoconstrictor peptide that mediates cell proliferation, fibrosis, and inflammation. ET-1 has 2 receptors A and B.

*Objectives: * The present study investigated whether administration of ET-1 receptor type A antagonist leads to protect cisplatin (CP) induced nephrotoxicity in ovariectomized-estradiol (Es) treated rats.

*Materials and Methods: * Thirty-six ovariectomized Wistar rats were divided into 6 groups. Group 1 received CP (2.5 mg/kg/day) for one week. Groups 2 and 3 received 2 different doses of Es (0.25 and 0.5 mg/kg/week) for 3 weeks, but CP was started in the third week. Group 4 was treated as group 1, but bosentan (BOS, 30 mg/kg/day) was also added. Groups 5 and 6 treated similar to groups 2 and 3 but CP and BOS were added in the third week. At the end of the experiment, blood samples were obtained, and the animals were sacrificed for histopathological investigation of kidney tissue.

*Results: * The serum levels of creatinine (Cr) and blood urea nitrogen (BUN) increased by CP; however, BOS significantly elevated the BUN and Cr levels that were increased by CP administration (*P* < 0.05). Co-treatment of Es, BOS, and CP decreased the serum levels of BUN, Cr, and malondialdehyde (MDA) when compared with the group treated with BOS plus CP (*P* < 0.05). Such finding was obtained for kidney tissue damage score (KTDS). As expected, Es significantly increased uterus weight (*P* < 0.05). The groups were not significantly different in terms of serum and kidney nitrite, kidney weight (KW), and bodyweight

*Conclusions:* According to our findings, BOS could not protect renal functions against CP-induced nephrotoxicity. In contrast, Es alone or accompanied with BOS could protect the kidney against CP-induced nephrotoxicity via reduction of BUN, Cr, and KTDS.

**Implication for health policy/practice/research/medical education:**

To find the role of endothelin receptor antagonist; bosentan (BOS) in cisplatin-induced nephrotoxicity in ovariectomized estradiol treated rats, we found, BOS could not protect renal functions against cisplatin-induced nephrotoxicity. In contrast, estradiol alone or accompanied with BOS could protect the kidney against cisplatin-induced nephrotoxicity by reduction of blood urea nitrogen (BUN), creatinine (Cr), and kidney tissue damage score.

## 1. Background

Cisplatin (cis-diamminedichloroplatinum II – CP) is known as one of the most successful drugs in cancer therapy (1). CP can be used for treatment of solid-organ cancers like testis, head and neck, ovary, cervix, and many other types of tumors (2). However, the major side effect of this drug is known to be nephrotoxicity, which is characterized by reduced glomerular filtration rate (GFR) and diminished renal blood flow (RBF). It is also accompanied by increased blood urea nitrogen (BUN) and creatinine (Cr) levels (3,4). CP causes tubular injury through multiple mechanisms, including hypoxia, generation of free radicals, inflammation, and apoptosis (5).

Clinically, CP-induced nephrotoxicity can be seen usually 10 days after treatment (2). It has been indicated that endothelin-1 (ET-1) plays a role in the pathophysiology of various forms of renal diseases, including renal failure (6), renal hypertension (7,8), and CP-induced nephrotoxicity (9). ET-1 is a potent renal vasoconstrictor that reduces both RBF and GFR (10). In fact, ET-1 have a more pronounced constrictive effect on renal artery than on coronary vessels. ET-1 also promotes mesangial cell growth and activates inflammatory cells (11).

Furthermore, upregulation of renal ET-1 receptors and overexpression of ET-1 gene have been reported in various animal models of acute renal injury (12). The ET-1 system receptors are ET-1 receptors A (ETAR) and B (ETBR). ETAR is abundantly expressed on vascular smooth muscle cells and cardiac myocytes and this receptor mediates the vasoconstrictory action of ET-1. This is while ETBR is predominantly expressed on endothelial cells where they stimulate the release of vasodilation and anti-proliferative mediators such as prostacyclin (13). ETBR stimulates both vasoconstriction and vasodilation (14) but under physiological conditions the predominant effect of the ETBR is a vasodilatory response (13). More recent studies using isolated human small pulmonary arteries suggested that a combined blockade of both ETAR and ETBR are necessary to achieve optimal inhibition of vasoconstriction (15,16).

Bosentan (BOS) is a nonselective dual ET-1 receptors antagonist. BOS produces vasodilatation of the vessels by acting as a competitive antagonist of ET-1 to both ETAR and ETBR (17). BOS is mainly metabolized in the liver and its metabolites are excreted in the bile (18). BOS has been shown to have some functions in acute lung injury and pulmonary fibrosis via three mechanisms; reducing the right ventricular systolic pressure, decreasing the release of reactive oxygen species, and relieving the inflammatory reaction (19). Some studies have investigated the effects of BOS on kidneys. ETAR and ETBR partially control RBF and tubular function (20), while RBF and GFR are decreased by CP (19). Therefore, it appears that BOS possibly can reduce CP-induced damages. Furthermore, our previous study indicated the estradiol (Es) has no beneficial effect against CP-induced nephrotoxicity (21).

## 2. Objectives

The current study was designed to investigate the effect of BOS on CP-induced nephrotoxicity in ovariectomized Es-treated rats.

## 3. Materials and Methods

### 3.1. Animals

Thirty-six adult female Wistar rats (189 ± 3.36 g) were handled in this study. All animals were maintained at a constant temperature of 25 ± 2°C with fixed 12:12-h light–dark cycle. Nutritionally balanced pellets and water were freely available.

### Experimental protocol

After anesthetizing by injection of ketamine (75 mg/kg, i.p.) the animals were ovariectomized as described previously (21). After recovery, they were randomly divided into 6 experimental groups. Group 1 received sesame oil (as solvent for Es) weekly for 3 weeks, and in the third week they also received CP (2.5 mg/kg/d, i.p.) + DMSO (solvent for BOS) on a daily basis. Groups 2 and 3 were treated with Es (0.25 and 0.5 mg/kg/wk, IM) for 3 weeks, and in the third week, they also received CP and DMSO as group 1.

Group 4 received CP, sesame oil, and BOS (30 mg/kg/d, i.p.) for 1 week. Groups 5 and 6 were treated as groups 2 and 3, respectively, but in the third week CP and BOS were added to the treatment.

At the end of the experiment (1 week after CP injection), blood samples were obtained via heart puncture under anesthetization, and the rats were sacrificed humanely. Kidneys and uterus were removed, weighed immediately, and the left kidney was prepared for histopathological procedures and right kidney was homogenized for the measurement.

### 3.2. Drugs

BOS, DMSO, estradiol valerate, and CP were purchased from Osvah Pharmaceutical Company (OSVE Tehran, Iran), Merck (Germany), Aburaihan Co. (Tehran, Iran) and (EBEWE Pharma Ges.m.b.H Australia), respectively.

### 3.3. Measurements

The bodyweight of the animals was recorded daily. The serum levels of Cr and BUN were estimated using a RA-1000 autoanalyzer (Technicon, Ireland) with Pars Azmoon (Tehran, Iran) kits.

Serum and tissue levels of nitrite (stable NO metabolite) were measured using a colorimetric assay that involves the Griess reaction. The renal and serum levels of malondialdehyde (MDA) were measured by thiobarbituric acid (TBA) 0.67% and trichloroacetic acid (TCA) 10% (22).

### 3.4. Histopathological procedures

The removed kidneys were fixed in formalin solution. Hematoxylin and eosin staining was applied to examine the tubular damage. The damage was evaluated by a pathologist who was totally blind to the study. Based on the intensity of tubular lesions (hyaline cast, debris, vacuolization, flattening and degeneration of tubular cells, and dilatation of tubular lumen), kidney tissue damage score (KTDS) was determined to be in the range of 1-4. Zero was considered for normal tissue.

### 3.5. Ethical issues

The research followed the tenets of the Declaration of Helsinki. The research was approved by ethical committee of Isfahan University of Medical Sciences. Prior to the experiment, the protocols were confirmed to be in accordance with the Guidelines of Animal Ethics Committee of Isfahan University of Medical Sciences.

### 3.6. Statistical analysis

Data are expressed as mean ± SEM. The levels of BUN, Cr, MDA, and nitrite; kidney weight (KW), and uterus weight were analyzed by the one-way analysis of variance (ANOVA) followed by the LSD test. The KTDS was compared by the Kruskal-Wallis or Mann-Whitney U tests. The *P* values ≤ 0.05 were considered statistically significant.

## 4. Results

### 4.1. Effects of BOS and Es on serum levels of BUN and Cr

CP alone increased the serum levels of BUN and Cr from normal levels. However, BOS not only did not attenuate these parameters toward normal value, but also significantly increased the serum levels of BUN and Cr in BOS + CP group in comparison with the CP alone treated group (*P* < 0.05). In addition, Es or Es plus BOS significantly reduced the serum levels of these parameters when compared with BOS + CP treated group (*P* < 0.05). These findings revealed that Es or Es +BOS could reduce the serum levels of BUN and Cr increased by CP alone.

### 4.2. Effects of BOS and Es on serum and kidney levels of MDA and nitrite

**Figure 1:**
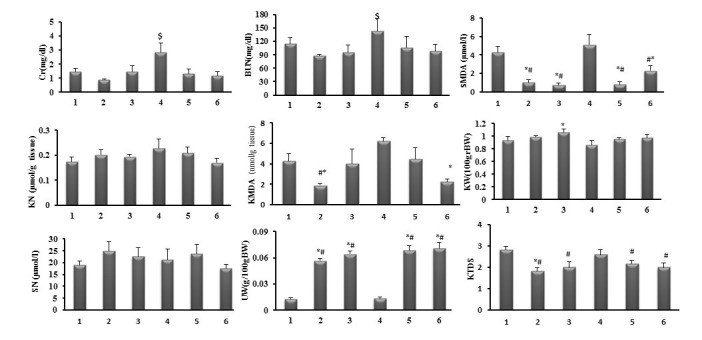
Biochemistry parameters measured in serum and kidney, uterus weight, body and kidney weights, and kidney tissue damage scores in the 6 experimental groups. The data is shown as mean ± SEM. $, *, and # indicate significant difference from the other groups, from group 4, and from group 1, respectively (*P *< 0.05). BUN: blood urea nitrogen; Cr: creatinine; SMDA: serum malondialdehyde; KMDA: kidney MDA; SN: serum nitrite; KN: kidney nitrite; US: uterus weight per 100 g bodyweight; KW: kidney weight per 100 g bodyweight; KTDS: kidney tissue damage score; group 1: CP (2.5 mg/kg/d), group 2: CP + ES (0.25 mg/kg/wk), group 3: CP + ES (0.5 mg/kg/wk), group 4: CP + BOS, group 5: CP + BOS + ES (0.25 mg/kg/wk), group 6: CP + BOS + ES (0. 5 mg/kg/wk).

The serum and kidney levels of MDA increased insignificantly in the BOS plus CP treated group (group 4) when compared with the CP alone treated (group 1). Moreover, Es either alone or accompanied with BOS significantly decreased the serum level of MDA when compared with CP alone or CP + BOS treated groups (*P* < 0.05). Similar observation was found for kidney tissue level of MDA when low dose of Es alone or high doses of Es+Bos were administered. Although Es increased the serum nitrite level insignificantly, the groups were not significantly different in kidney tissue and serum levels of nitrite (Figure 1).

### 4.3. Effects of BOS and Es on uterus, kidney, and body weights

**Figure 2:**
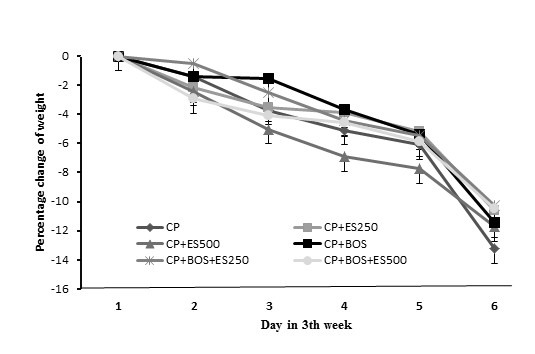
Percentage of bodyweight changes in all experimental groups during cisplatin (CP) therapy.

As expected, uterus weight was significantly increased in all Es-treated groups when compared with the non-Es groups (*P* < 0.05). Bodyweight was decreased in all groups significantly (*P* < 0.05) with no statistical differences among the groups. Finally, BOS in BOS plus CP treated group did not alter the KW when compared with the CP alone treated group (Figure 2).

### 4.4. Effects of BOS and Es on KTDS

**Figure 3:**
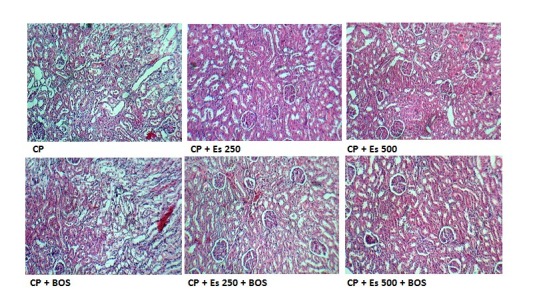
Sample images of kidney tissue. The tissue damage is lowered when bosentan (BOS) was accompanied with estradiol.

CP alone and accompanied with BOS increased renal damage insignificantly but KTDS in Es or Es plus BOS groups decreased significantly when compared with the CP group (*P* < 0.05). This factor was reduced insignificantly in the Es + BOS treated group compared with the CP + BOS treated group. The sample images of kidney tissues are demonstrated in Figure 3.

## 5. Discussion

The main objective of this study was to investigate the effect of BOS on CP-induced nephrotoxicity in Es-treated ovariectomized rats. Our findings supported that vasodilatory effect of BOS did not improve the kidney damage against CP, but Es or Es + BOS may improve it. Coa et al reported that administration of BOS did not improve renal injury in subtotally nephrectomized animal model, and it did not decrease plasma urea and Cr concentrations. Also, they indicated ET-1 antagonist was ineffective in lowering blood pressure, decreasing proteinuria, or slowing the decline in GFR in subtotally nephrectomized rats. Other investigators also found some adverse effects of BOS (23,24). It is reported ETAR and ETBR distributed differently in the kidney and both receptors are present on glomerulus (25,26), and BOS exhibits a relative ETA:ETB affinity of 20:1 (27). So, it is possible that BOS blocks vasoconstrictory effect more than the vasodilatory response. Consequently, the prominent effect is vasodilation and CP could enter more into the kidney. BOS also can increase plasma levels of ET-1 in pigs (27,28). Therefore, the expected protective role of BOS may be reverse in CP treated rats. In addition, we previously administered CP as single dose and no beneficial effect of Es against CP-induced nephrotoxicity was reported (29) while some studies have reported its useful effects including the antioxidant properties and formation of vasodilator biomarkers such as NO (30,31). Polderman et al (32) reported that females have lower ET-1 levels and this was reversed in ovariectomized rats (33,34). Also, it is found that ovarian hormones suppress ET-1 production and Es could attenuate contractile effects (35). Rodrigo et al showed vascular ET converting enzyme-1 (ECE-1) expression was decreased by Es in mesenteric arteries (36). In addition, CP alone and CP plus BOS treated groups were ovariectomized and the Es source was removed. In this case, higher damages in these groups duo to increased ET-1 by BOS and absence of Es was expected. Collectively, these data suggest that administration Es probably decreased ECE-1 and ET-1 expression. Also, it was indicated that Es decreased the plasma urea and Cr and MDA levels in kidney in diabetic ovariectomized rats (37). Hekimoglu et al (38) showed that Es treatment decrease NO and MDA levels in ovariectomized rats. It was also reported that Es treatment decline Cr and urea concentrations, vascular damage index, the protein expression of TGF-β and collagen IV, and expression of both ET-1 receptors (ETA and ETB) in uninephrectomized SHRsp rats (39). As mentioned above, it is possible that the presence of Es improved renal insufficiency when it is accompanied with CP and CP plus BOS. Uterus weight in our study increased in Es treatment groups. The evidence available demonstrates that uterus weight increase with administration of different doses of Es (0-500 µg/kg/day) in ovariectomized rats (40). Finally, bodyweight loss was observed in CP plus BOS group and Es recovered it. Seibold et al (41) reported BOS can cause clinical problems like diarrhea, peripheral edema, and anemia; and administration of CP alone causes gastrointestinal disorders (42).

## 6. Conclusions

It is concluded that BOS could not protect kidney from CP-induced nephrotoxicity but when it is accompanied with ES, it may improve the nephrotoxicity.

## 7. Limitations of the study

The main limitation of the current study was related to estradiol administration. Estradiol was given as single dose in each week. It was preferred to have estradiol releasing in slow model such as transdermal daily dose model.

## Authors’ contribution

AZ; run the experiment, preparation of manuscript and data analysis. MN; study design, preparation of manuscript, final revision, and data interpretation. MM; run the experiment. AT; pathology investigation.

## Acknowledgments

A part of this study was presented in the International Congress of Nephrology and Urology (ICNU) 2015, in Tehran.

## Conflicts of interest

The authors declared no competing interests.

## Funding/Support

This research was supported by Isfahan University of Medical Sciences (grant # 293111)
